# Hollow Fiber Porous Nanocomposite Membranes Produced via Continuous Extrusion: Morphology and Gas Transport Properties

**DOI:** 10.3390/ma11112311

**Published:** 2018-11-17

**Authors:** Zahir Razzaz, Denis Rodrigue

**Affiliations:** CREPEC (Research Centre for High Performance Polymer and Composite Systems), CQMF (Quebec Centre on Functional Materials), Department of Chemical Engineering, Université Laval, Quebec, QC G1V 0A6, Canada; zahir.razzaz.1@ulaval.ca

**Keywords:** polyethylene, hollow fiber, mixed matrix membranes, gas separation, extrusion, cellular structure

## Abstract

In this work, hollow fiber porous nanocomposite membranes were successfully prepared by the incorporation of a porous nanoparticle (zeolite 5A) into a blend of linear low-density polyethylene (LLDPE)/low-density polyethylene (LDPE) combined with azodicarbonamide as a chemical blowing agent (CBA). Processing was performed via continuous extrusion using a twin-screw extruder coupled with a calendaring system. The process was firstly optimized in terms of extrusion and post-extrusion conditions, as well as formulation to obtain a good cellular structure (uniform cell size distribution and high cell density). Scanning electron microscopy (SEM) was used to determine the cellular structure as well as nanoparticle dispersion. Then, the samples were characterized in terms of mechanical and thermal stability via tensile tests and thermogravimetric analysis (TGA), as well as differential scanning calorimetry (DSC). The results showed that the zeolite nanoparticles were able to act as effective nucleating agents during the foaming process. However, the optimum nanoparticle content was strongly related to the foaming conditions. Finally, the membrane separation performances were investigated for different gases (CO_2_, CH_4_, N_2_, O_2_, and H_2_) showing that the incorporation of porous zeolite significantly improved the gas transport properties of semi-crystalline polyolefin membranes due to lower cell wall thickness (controlling permeability) and improved separation properties (controlling selectivity). These results show that mixed matrix membranes (MMMs) can be cost-effective, easy to process, and efficient in terms of processing rate, especially for the petroleum industry where H_2_/CH_4_ and H_2_/N_2_ separation/purification are important for hydrogen recovery.

## 1. Introduction

Microporous polymer membranes are currently considered as commercially attractive due to their low operating temperature and manufacturing costs, as well as good processability. However, due to limited polymer thermal stability and the plasticization effect, their applications have been restricted to separation processes where severe conditions are not encountered [1,2]. One effective approach to improve the performance of polymer membranes is to incorporate inorganic nanoparticles such as zeolites and carbon-based molecular sieves since they have higher thermal resistance and chemical stability, combined with molecular sieving property. This led to the concept of mixed matrix membranes (MMMs) which were shown to have increased selectivity and permeability compared to neat polymer membranes [3,4]. Nevertheless, the addition of these inorganic particles makes the membranes more fragile.

A large number of investigations focused on membrane production to improve the gas transport properties based on material selection, especially for commercial-scale applications. However, very few studies focused on the control of the morphological structure in relation with the membranes’ performances [5,6,7].

Several methods are available to prepare asymmetric and symmetric porous polymer membranes, but most of them are based on solvent casting followed by phase separation [8], where solvent toxicity and costs (recycling/elimination/treatment) are the main drawbacks of these non-environmentally friendly methods. On the other hand, a few methods have been proposed to create a microporous membrane without any solvents. The use of leachable particles such as salts was shown to produce an open-cell structure based on polystyrene [9], polypropylene [10], and polyethylene [11]. Other solvent-free methods are stretching with or without particles [12] and direct foaming of semi-crystalline thermoplastics such as high-density polyethylene (HDPE) and polypropylene (PP) [7,13]. For stretching methods, the porosity is created by delamination between the matrix and local stress concentration points such as solid particles or crystalline zones, but thermal post-treatment is needed to stabilize the newly created microporous and crystalline structure to avoid shrinkage and warpage, which are the main drawbacks of such methods.

However, the cellular structure of a polymer can be produced via direct foaming processes leading to weight and waste reduction, heat resistance, high strength to weight ratio, low cost, recyclability, multi-functionally, as well as easier processing. Today, polymer nanocomposite foams can be produced via extrusion [14], injection molding [15], compression molding [16], in situ polymerization [17], and phase separation [18]. Extrusion is however the only one being continuous. Thus, extrusion is very effective, fast, and economic to produce low-density foams [19,20]. The cellular structure can be produced using either chemical or physical blowing agents (PBAs), but chemical blowing agents (CBAs) are generally easier to handle with standard equipment compared to physical blowing agents [7,21].

The authors’ previous works showed how the morphological properties, such as cell size uniformity as well as cell density, can control the physical, mechanical and gas transport properties of hollow fiber polymer membranes [5,6,7,13]. Since these properties are tunable, this led to membrane performance enhancement via simultaneous improvement of both the surface area as well as gas transport properties [7,22]. Then, a melt processing method was optimized to produce hollow fiber foamed membranes (HFM) based on a microcellular blend of linear low-density polyethylene (LLDPE) and low-density polyethylene (LDPE) to get a balance between foamability and mechanical strength. Furthermore, the blowing agent content, processing (temperature profile, flow rate, screw speed, etc.), and post-processing (stretching) conditions were the main parameters to control the porous membrane structure. Direct foaming is interesting because it is solvent free, continuous, and fast to produce a porous structure in hollow fibers [5,7,13]. This configuration is the most interesting because of its higher active surface area per unit volume compared with flat membranes.

Among a wide range of resins, polyolefins such as polyethylene (PE) are today one of the most widely used thermoplastic resins due to their excellent overall performance and thermomechanical properties, chemical resistance, and low cost, as well as easy processability and recyclability. Polyethylene membranes have been commonly used in microfiltration and blood oxygenation applications [11,23,24,25], but their application as gas separation membranes has been limited because of their low gas transport properties owing to their semi-crystalline nature. However, this can be modified. For example, Covarrubias and Quijada [26] investigated the effect of aluminophosphate (ALPO) addition into PE membranes through melt compounding. The results showed that a substantial improvement in gas transport properties was obtained by the creation of a gas transport pathway provided via the swollen ALPO. Hydrogen permeability was increased from 1.4 to 11.9 Barrers and H_2_/CO selectivity increased from 1.2 to 17. Nevertheless, polyolefin-based composite membranes for gas separation have barely been investigated, and this lack of information limits their use since the relation between membrane performances and morphology is not well understood. This is why one of the objectives of this work is to investigate the effect of foaming conditions on cell density and cell size distribution by the addition of nanoparticles to control not only the mechanical, but also the gas permeation properties of these materials. However, the foaming of semi-crystalline polymers, such as polyethylene, is more challenging compared to amorphous resins, since particle addition has a direct effect on foam cell nucleation and crystal nucleation. These resins are also known to have a very narrow foaming temperature window [27,28,29].

Nevertheless, the authors’ previous results showed that hollow fiber membranes based on foamed LLDPE/LDPE blends can be a good starting point due to their interesting gas permselectivity. Although several approaches are available to improve the foamability of a polymer [27,30], it was found that nanoparticle addition as a nucleating agent can be a simple but very effective strategy to improve the cellular structure of polymer foams in terms of cell density and cell size uniformity [31,32]. Two different types of nucleation mechanisms are known (homogeneous and heterogeneous) [32], but heterogeneous nucleation becomes predominant when particles with a high surface area are used [33]. This approach has been successfully applied for polyolefins using Ca_2_CO_3_ [14], clay [34], silica [6,21,35], and Fe_3_O_4_ (OA-Fe_3_O_4_) [36].

To improve membrane properties, different particles have been used. The focus for this study was the zeolite nanoparticle [7,13]. However, the main issues in matrix membrane production are particle spatial distribution (agglomeration) [6], as well as possible interfacial rigidification and pore blockage [37]. For this reason, zeolite nanoparticles were added through a well-controlled extrusion process to limit these issues.

This work is also a second step to improving upon the results obtained from the authors’ previous studies [6,7,13]. In fact, the idea was to propose a sustainable alternative production method for hollow fiber mixed matrix membranes. The matrix was made from foamed LLDPE/LDPE (70/30) and the addition of zeolite as nucleation agent/gas permeation modifier was performed to investigate its effects on the cellular structure and gas separation performance of the resulting nanocomposite foams referred to as mixed matrix foam membranes (MMFMs). The membranes were prepared through a continuous non-solvent method (foam extrusion) with a focus on zeolite content. For the separation performances, permeability and ideal selectivity were determined.

## 2. Materials and Methods

### 2.1. Materials

The material used in this study was linear low-density polyethylene (LLDPE) powder LL 8460 with a melting temperature of 126 °C, a density of 0.938 g/cm^3^, and a melt index of 3.3 g/10 min (190 °C at 2.16 kg) provided by ExxonMobil (Calgary, AB, Canada). The low-density polyethylene (LDPE) used was LA-0219-A supplied by Nova Chemicals (Calgary, AB, Canada). It has a melt flow index (MFI) of 2.3 g/10 min (190 °C at 2.16 kg) and a density of 0.939 g/cm^3^. As a chemical blowing agent (CBA), activated azodicarbonamide (Celogen 754A) was purchased from ChemPoint (Midland, MI, USA). According to the information from the supplier, this CBA has a gas released of 200 cm^3^/g of a mixture of N_2_, CO_2_, CO, and NH_3_. Its decomposition takes place between 165–180 °C. Zeolite 5A nanoparticles were purchased from Sigma-Aldrich (Oakville, ON, Canada) and used as received. These nanoparticles have a size less than 3 µm. The permeation performance of each sample was performed by using commercially important gases having various kinetic diameters, including H_2_ (2.89 Å), CO_2_ (3.30 Å), O_2_ (3.46 Å), N_2_ (3.64 Å), and CH_4_ (3.80 Å).

### 2.2. Sample Preparation

**Figure 1:**
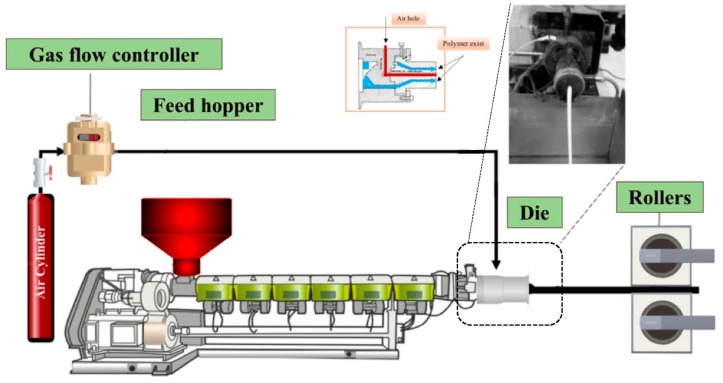
Schematic representation of the extrusion set-up to produce hollow fiber mixed matrix foamed membranes.

Two blending techniques were used to produce a cellular structure with a LLDPE/LDPE (70/30) blend with different zeolite nanoparticles contents (10, 15, and 20 wt %) and an optimum chemical blowing agent content (2.5 wt %). Before blending, both polymers, CBA, and zeolite were dried at 70 °C for 24 h. In the first approach, LLDPE/LDPE/zeolite compounds were prepared by melt compounding (extrusion) at 140 °C and 50 rpm. The process was done on a co-rotating twin-screw extruder (ZSE-27, Leistritz, Nürnberg, Germany) with a length/diameter (L/D) of 40 and a diameter of 27 mm with 10 individually heated/cooled zones. Further details for the optimum base conditions can be obtained in the authors’ previous study [13]. In the second approach, since all the materials were in a powder form (both polymers, zeolite, and CBA), they were simply dry-blended for 20 min. For foaming, the same extruder as for melt compounding was used, but a tubular die (inside diameter of 3.5 mm and outside diameter of 5 mm) was used instead of a circular die and the screw speed was decreased to 25 rpm. At the extruder’s exit, the material was introduced in a two-roll calendaring system to impose different drawing speeds and have a control on the stretching (post-extrusion) extrusion. The complete experimental set-up is shown in Figure 1. The foamed membranes prepared by using direct foaming are referred to as mixed matrix foamed membrane (MMFM) followed by a number showing the zeolite content (wt %). For comparison, unfoamed (compact) samples have also been produced with the same zeolite contents using the same processing conditions. These samples (L) are named as L-0, L-10, L-15, and L-20 where the number refers to the zeolite content. More details can be found in Table 1.

### 2.3. Characterization

Scanning electron microscopy (SEM) was performed on a JSM 840A (JEOL, Tokyo, Japan) to analyze the zeolite dispersion and foam structure. For each sample, images were taken in two perpendicular directions, namely, the flow (F) and transverse (T) directions, to get a complete 3D evaluation of the structure related to deformation in the stretching direction. The samples were cut with a doctor blade and the exposed surfaces were made conductive by deposition of a thin gold/palladium coating. Cell density and cell size were determined by using the Image J software (National Institutes of Health, Bethesda, MD, USA). The values are reported as an average of at least three images in each direction. Due to cell deformation, the cell density (*N*) was determined as [38]:

(1) N=N1(N2)12

where *N_1_* and *N_2_* are defined as the surface cell densities in the F and T directions, respectively, and determined as:

(2) $$N_{i}=\frac{n}{A}$$

where *n* is the number of cells in a defined area *A* (cm^2^).

Density was obtained by using a gas (nitrogen) pycnometer Ultrapyc 1200^e^ (Quantachrome, Boynton Beach, FL, USA). Thermogravimetric analysis (TGA) was used to determine the weight loss curves and evaluate the samples’ thermal stability as well as to confirm the particle contents. This was done on a Q5000IR (TA Instruments, New Castle, DE, USA) from 50 to 800 °C at a heating rate of 10 °C/min under nitrogen. The matrix crystallinity was determined via differential scanning calorimetry (DSC) on a DSC 7 from Perkin Elmer (Waltham, MA, USA). The experiments were conducted using approximately 5 mg of samples in aluminum pans under N_2_ at a rate of 10 °C/min. A heating/cooling/heating between 50 to 200 °C was used to run the experiments. The results extracted from the first heating cycle were noted to show the influence of foaming history on the crystallization degree of the polymer blend/nanofiller foamed composites. The mechanical properties were obtained with a 500 N load cell and a 10 mm/min crosshead speed at ambient temperature (23 °C) using a universal testing machine model 5565 (Instron, Norwood, MA, USA). To report on the Young’s modulus, tensile strength, and elongation at break, a minimum of five replicates was used.

### 2.4. Gas Permeation

**Figure 2:**
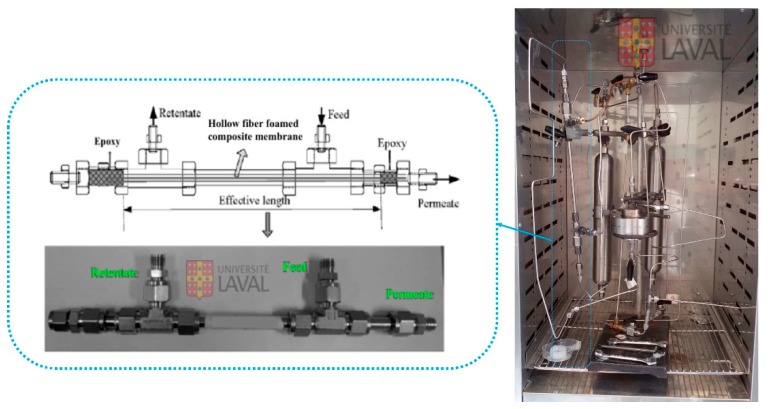
Schematic representation of the (**right**) permeation set-up with the (**left**) hollow fiber membrane module set-up.

The separation performance of the membranes was measured by gas permeation analysis. As presented in Figure 2, a sample was fixed in a permeation hollow fiber module. The module was evacuated (feed and permeate sides) for a minimum of 6 h under vacuum. To start the experiment, a gas was introduced on the feed side at a pressure 30 psi, while the permeate side had a constant volume. Then, the pressure variation on the permeate side was measured as a function of time until a steady state was achieved, which was used to determine the permeance by the solution-diffusion model. All the tests were carried out at 30 °C. The values reported are the average of at least five measurements and the permeance coefficient (*Q* in gas permeation unit (GPU)) was determined as follows:

(3) $$Q=\left(\frac{P}{l}\right)=\frac{22414}{A}\times \frac{V}{RT\Delta p}\times \frac{dp}{dt},$$

where *V* is the constant volume of the permeate side (cm^3^) and *L* and *A* are membrane thickness (cm) and the membrane area (cm^2^), respectively. $\frac{dp}{dt}$ is the rate of permeation in the constant volume under steady state condition (psi/s), ∆*p* is the variation between the upstream pressure and permeate pressure, *T* is the absolute temperature (K), and *R* is the universal gas constant (6236.56 cm^3^ cmHg/mol K). The ideal selectivity between two gases A and B is defined as the ratio between the more permeable gas (A) and the less permeable one (B), as follows:

(4) $$\alpha_{A/B}=\frac{Q_{A}}{Q_{B}}$$

## 3. Results and Discussion

### 3.1. Preparation and Optimization of MMFMs

The foaming of polyolefins, such as polyethylene, is generally more challenging in comparison with most polymers (such as polystyrene) due to a quite narrow foaming temperature window for semi-crystalline polymers [19,32]. In the authors’ previous study, it was shown that a cellular structure of LLDPE/LDPE (70/30) having a high cell density (1.7 × 10^7^ cells/cm^3^) and a uniform cell size distribution could enhance the membrane permeance and selectivity by approximately 100% and 75%, respectively, in comparison with a compact (unfoamed) LLDPE/LDPE membrane [13]. The current work confirms their previous findings and provides additional evidence suggesting that zeolite addition improved these properties. Multiphase polymer materials, in comparison with neat polymers, can have a different foaming behavior because of low-energy interfacial regions. It is known that using a polymer with a low melt strength for foaming leads to poor cell morphology due to cell rupture/coalescence resulting from poor cell wall elasticity associated with biaxial elongational flow around growing cells. Moreover, to improve the matrix (LLDPE/LDPE) melt strength and its nucleation behavior, particle addition (nucleating agent) has been reported [21,32,39].

### 3.2. Morphology

The dispersion degree of nanoparticles in the polymer matrix has a considerable effect on the final results [30]. In this study, the zeolite dispersion degree was even more critical to control the gas permeation. An excellent dispersion has a positive nucleating effect, whereas particle agglomeration decreases the effectiveness of these nanoparticles as bubble/crystal nucleators during the foaming process. They can also create non-selective channels for the gas molecules leading to poor separation performance [40,41,42].

**Figure 3:**
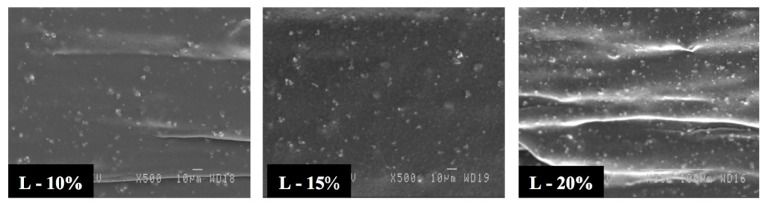
High-resolution images showing zeolite nanoparticle dispersion in the matrix.

Firstly, Figure 3 presents typical images for the unfoamed nanocomposites. The bright white spots are zeolite nanoparticles where well-dispersed aggregates of approximately 1 to 3 µm are seen. It is also clear that the aggregate number increases with zeolite content. These results are in agreement with other studies performed on polyethylene as the matrix and zeolite nanoparticles with larger diameters [43,44].

**Figure 4:**
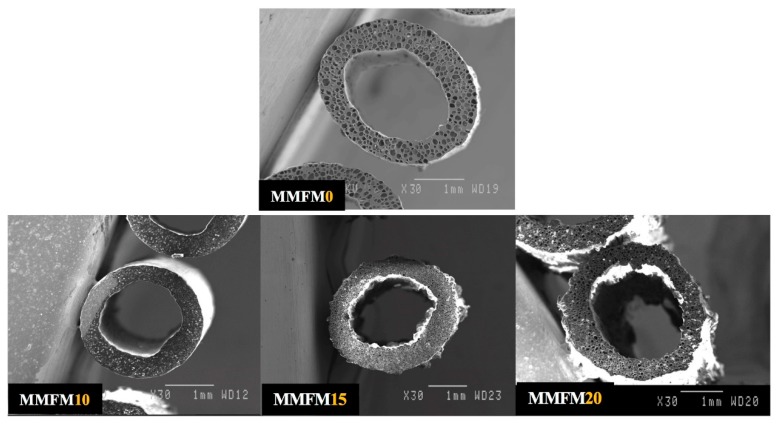
Micrographs of the hollow fiber foam membranes with different zeolite contents (0, 10, 15, and 20 wt %) at a stretching speed of 5 m/min.

**Figure 5:**
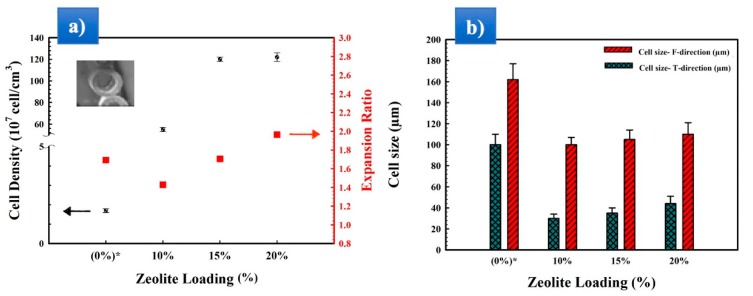
(**a**) Cell density and expansion ratio, (**b**) average cell sizes in the transverse (T) and flow (F) directions of mixed matrix foam membrane (MMFMs) with different zeolite contents (0, 10, 15, and 20 wt %) at a stretching speed of 5 m/min.

Figure 4 presents typical cross-section images for MMFMs with different zeolite particles loading (0, 10, 15, and 20 wt %) using a stretching speed of 5 m/min. From the micrographs, it can be observed that the addition of the porous zeolite particles produced a significant increase in the number of cells and a reduction in cell size. Based on the images taken, Figure 5a presents the cell density as a function of zeolite content for a die temperature of 168 °C and a stretching speed of 5 m/min. These results show that with zeolite addition, the cell density significantly increased (two orders of magnitude) from 1.2 × 10^7^ cells/cm^3^ for MMFM0 to 120 × 10^7^ cells/cm^3^ for MMFM15. A negligible variation was observed at higher zeolite content (20 wt %), probably due to severe particle agglomeration.

On the other hand, the expansion ratio did not change much (1.4–1.9) between all the samples studied. Nonetheless, the effect of zeolite on the average cell size (F and T directions) is clearly seen in Figure 5b. The results reveal a significant cell size reduction in the transverse direction compared to the neat foam (0% zeolite). For example, the average cell size in the T direction decreased from 105 to 30 µm at 10% zeolite. Then, a slight increasing is observed with increasing zeolite content. These results are consistent with those reported for a LDPE/silicon system [21,35]. Similarly, filler addition decreased the average cell size in the flow direction from 165 to 110 µm. The optimum zeolite content may be related to gas loss and cell coalescence when the zeolite content is too high (Figure 4). Finally, based on the above findings, the reason for the enhanced foaming potential of LLDPE/LDPE/zeolite blends is the presence of zeolite, leading to increased heterogeneous nucleation. Also, it should be mentioned that samples with less than 10% of zeolite did not show a significant increase in those parameters, the reason for which the findings are not presented.

### 3.3. Effect of Blending Method on the Cellular Structure

**Figure 6:**
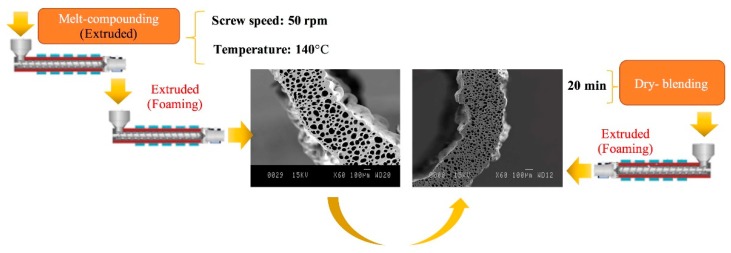
Effect of the production method on the MMFM morphology with 15 wt % zeolite: (**left**) melt compounding and (**right**) dry blending.

**Figure 7:**
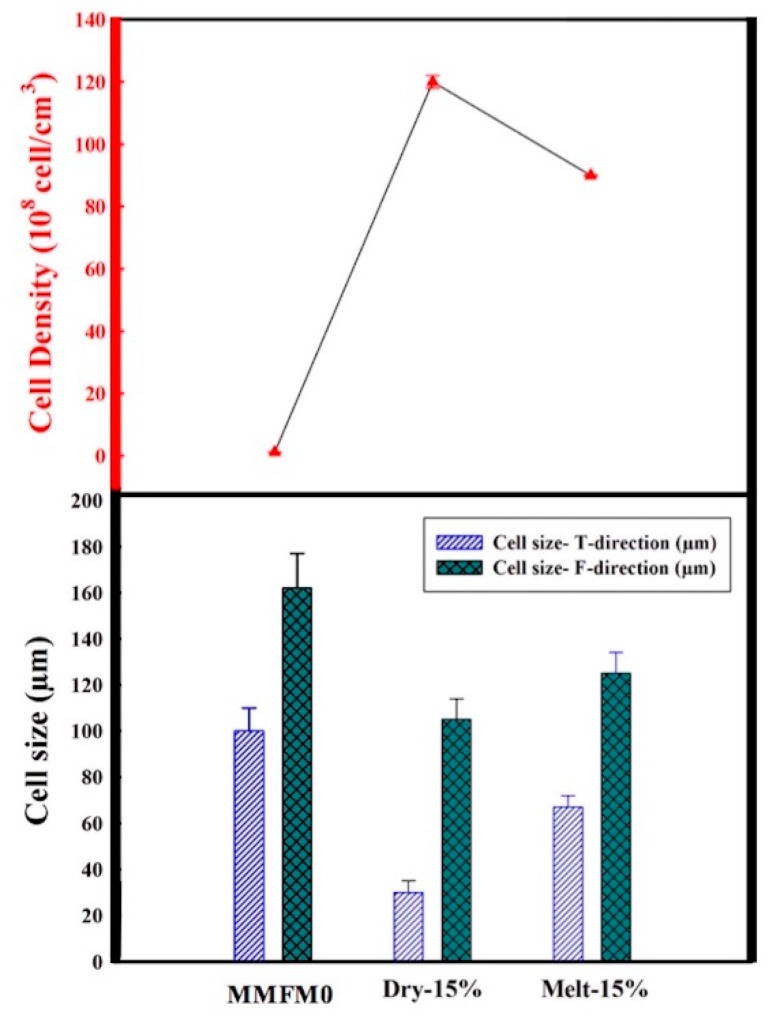
Effect of blending method (melt compounding and dry blending) on the cell size in both T and F directions, as well as cell density for MMFM0 and MMFM15.

Before the foaming process, two methods were used to introduce the zeolite in the polymer matrix, namely, melt compounding and dry blending. Figure 6 presents typical results for the sample MMFM15. Interestingly, samples based on dry blending led to lower cell sizes compared to melt compounding. Figure 7 reports on the results after image analysis. The cell size reduction is assumed to be related to the possible absorption of gases which are released from the CBA by the porous zeolite nanoparticles. As melt compounding may lead to possible pore blockage and/or particle collapse/shrinkage, more gas is available and released for cell growth compared with dry blending. This effect is more important as zeolite content increases since a higher cell density is obtained at a higher zeolite concentration (10–20 wt %) with 2.5% CBA. Based on this observation, it is expected that the addition of porous zeolite nanoparticles can lead to lower cell sizes, especially using a dry blending method before foaming.

**Figure 8:**
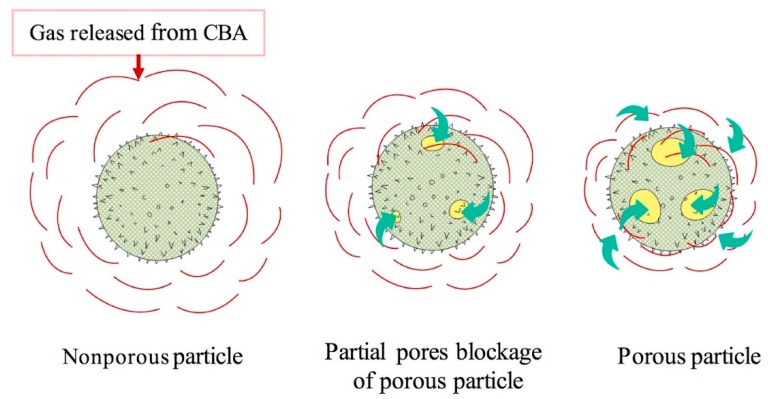
Schematic representation of the porous and nonporous nucleating agent mechanism of zeolite during foaming.

The effect of zeolite on PE foam nucleation is thus affected by the blending method, and a schematic representation in presented in Figure 8 to explain the mechanisms. The energy barrier associated with homogeneous nucleation is usually much higher compared to heterogeneous nucleation [45]. Consequently, the surface of a nucleating agent has lower surface free energy (energy barrier) and the particles act as heterogeneous nucleation points leading to higher cell density and smaller cell sizes. Interestingly, there were negligible differences in the foam density with zeolite addition. However, zeolite addition via dry blending prior to foaming seems to provide a higher surface area because of more available open pores due to the gas cavities. Therefore, the foams have higher cell density and smaller cell size. This finding is in agreement with recent results on poly(methylmethacrylate) (PMMA) foams using nonporous (compact) (solid silica) and porous particles (spherical ordered mesoporous silica) as nucleating agents [46]. This effect will be further discussed in the permeation section.

### 3.4. Effect of the Stretching Rate on the Cellular Structure

**Figure 9:**
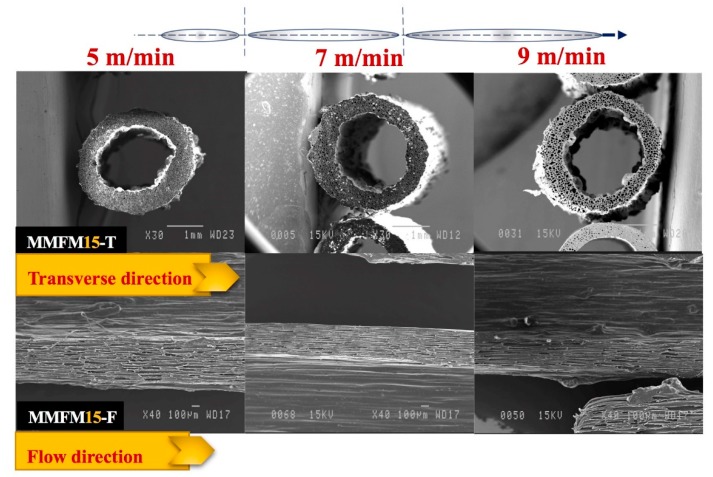
Micrographs of MMFM15 produced at different stretching speeds (m/min) for the (**top row**) transverse (T) and (**bottom row**) flow (F) directions.

**Figure 10:**
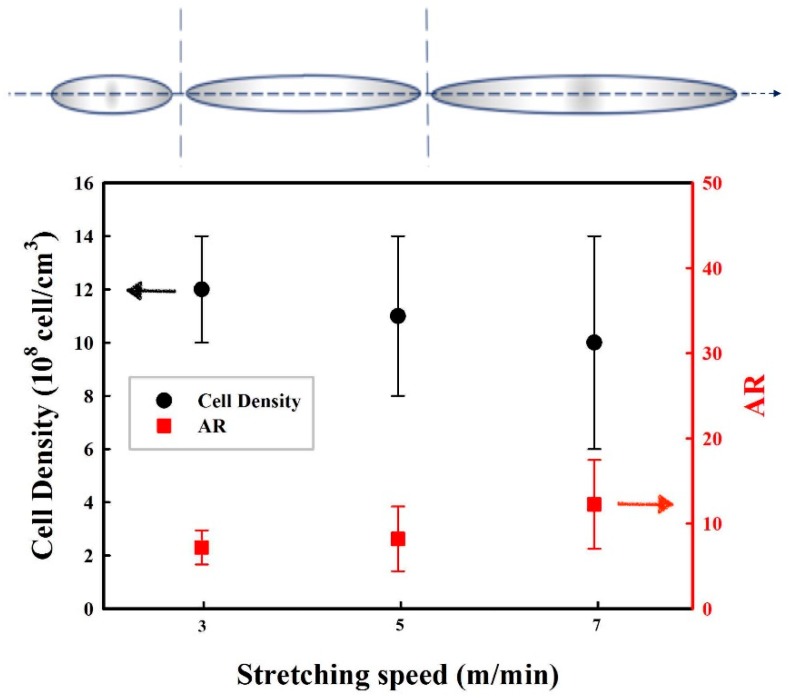
Effect of the stretching speed on cell density and cell aspect ratio (AR) in the flow direction for MMFM15 (2.5% CBA).

Figure 9 presents typical SEM micrographs in the flow (F) and transverse (T) directions of MMFM15 produced with 2.5% CBA at a constant die temperature (168 °C) and different stretching speeds (5, 7, and 9 m/min). As expected, based on the authors’ previous investigations, by increasing the stretching speed from 5 to 9 m/min more elongated cells in the flow direction are produced. Consequently, the cell aspect ratio increased with the stretching rate, as presented in Figure 10. Based on the image analysis, it can be observed that the cell density might slightly decrease with increasing stretching speed, but the variation may not be significant due to large distributions. However, the further increase in stretching speed (9 m/min) resulted in several cell wall ruptures and voids were created around the nanoparticle cellular structure. This is different than what was reported in the authors’ previous investigation where stretching speeds up to 13 m/min were used. This was possible because an unfilled PE matrix was used (no nanoparticles). In this study, interfacial voids around the nanoparticles were created because of delamination due to high stretching speed [12,43]. Therefore, an optimum stretching speed must be selected to get the best foam structure in terms of high cell density and narrow cell size distribution without any defects in the polymer structure for good membrane performances (i.e., no short-circuit).

### 3.5. Gas Permeation Performances

**Figure 11:**
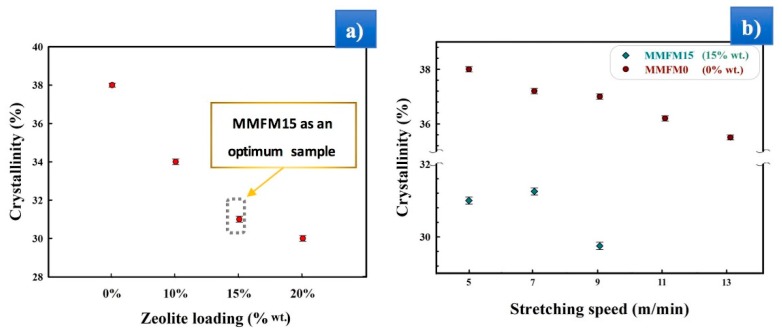
Effect of (**a**) zeolite loading and (**b**) stretching speed on the crystallinity of MMFMs.

The gas transport properties of polyolefins, as well-known semi-crystalline polymers, are a result of their crystallinity. Their crystalline regions can behave as gas barriers (regions of negligible diffusion and solubility), hence the penetrant gas molecules have a more tortuous path (diffusion path) [26,47,48]. Therefore, the permeability should overall decrease with an increasing degree of crystallinity, especially for larger gas molecules [49,50]. This can also be explained through the limitation of polymer chain mobility in the crystalline areas [7,48]. Overall, the size of the gas molecules can influence their mobility inside the polymer structure. Polyethylene, with a rubbery character, can also favor the condensation of gases in its free volume developed by the mobile and flexible chain molecules [26,51]. The permeability in porous dense polymer membranes is defined through the solution-diffusion model [6]. Thus, the effect of zeolite content on the film crystallinity is presented in Figure 11a. It can be seen that the crystallinity of the authors’ PE blend/zeolite foamed composites decreased as zeolite content increased, and similar results have been reported for PE above 10 wt % zeolite [43,44]. Generally, the introduction of inorganic particles not only replaces polymer crystals and occupies the crystal structure sites, but also creates more amorphous areas, especially at higher particle concentration [34,52,53].

**Figure 12:**
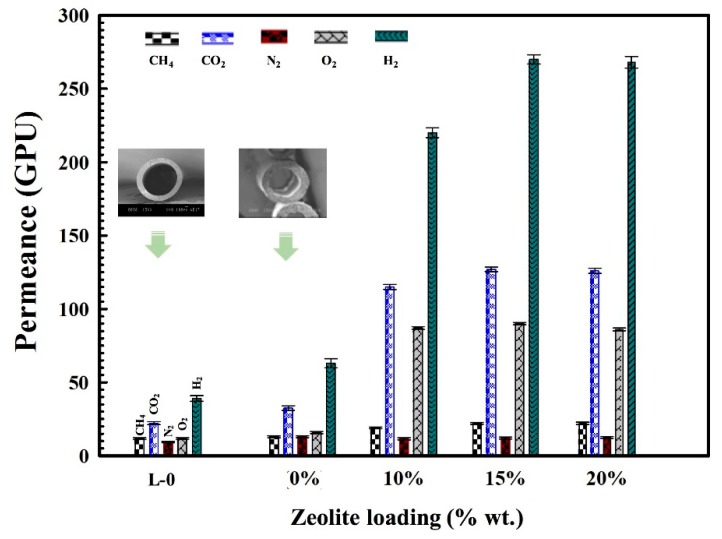
Permeance of various gases (CH_4_, CO_2_, N_2_, O_2_, and H_2_) at 30 °C and 30 psia for foamed and unfoamed hollow fiber MMFMs produced with different zeolite loading (0, 10, 15, and 20 wt %) at a stretching speed of 5 m/min.

The permeance (Equation (3)) of the MMFMs with different zeolite content is presented in Figure 12. The results show that the permeance significantly increases with zeolite addition compared to hollow fibers based on foamed and unfoamed MMFM0 membranes for all the gases studied. It was also found that the permeance increases with increasing zeolite concentration up to MMFM15 and then slightly decreases for MMFM20. This optimum content represents again a balance between the cell morphology created (cell size and cell density), the PE crystallinity level and the zeolite dispersion (see Figure 5a).

**Figure 13:**
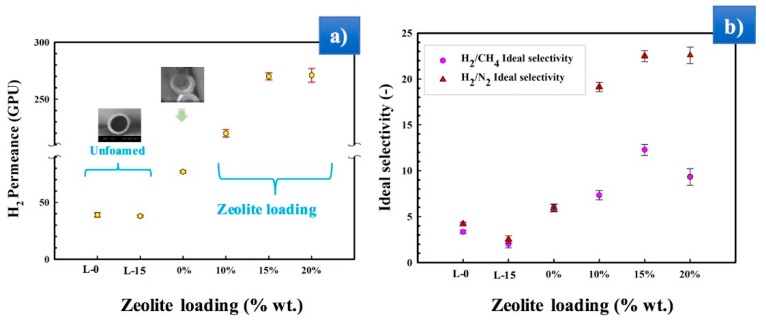
Permeation results for (**a**) H_2_ gas permeance and (**b**) H_2_/CH_4_ and H_2_/N_2_ ideal selectivity at 30 °C and 30 psia for different membranes as a function of zeolite loading.

However, the gas permeance of the foamed hollow fiber MMFM0 membranes is higher than the unfoamed (L-0) membranes for all the gases analyzed (H_2_, O_2_, CH_4_, CO_2_, and N_2_). The incorporation of zeolite nanoparticles within the polymer matrix produced a significant increase in the gas transport properties due to their porous nature. As expected, H_2_ permeance is the highest because of its smaller molecular size (2.98 Å) compared with other gases (O_2_: 3.46 Å, CO_2_: 3.30 Å, N_2_: 3.64 Å, and CH_4_: 3.80 Å). This also explains the higher permeance of CO_2_ compared to CH_4_, O_2_, and N_2_, especially due to its high condensability leading to higher solubility in polyethylene. In particular, Figure 13a shows that the H_2_ permeance increased by seven and three times for MMFM15 compared to the unfoamed (L-0) and foamed (MMFM0) membranes, respectively. This result can be related to more gas diffusion pathways provided by the porous nature of zeolites [42] and a larger number of voids in the foam structure [5,7,13].

The ideal selectivity (Equation (4)) results for H_2_/CH_4_ and H_2_/N_2_ are presented in Figure 13b. A negligible variation was observed in the unfoamed L-0 and L-15 samples for both values, but slight improvements were observed for the foamed membranes (MMFM0). However, all MMFMs exhibited superior H_2_/CH_4_ and H_2_/N_2_ ideal selectivity compared to samples without zeolite (MMFM0). At the optimum zeolite loading (15 wt %), the highest H_2_/CH_4_ ideal selectivity was 12.5, whereas the ideal selectivity for H_2_/N_2_ increased from 3.9 to 22.9. As mentioned above, since the small molecular size of H_2_ leads to a higher permeance than for the other gases, any membrane permeance improvement mostly favors H_2_ permeation. However, the main idea in porous zeolite addition is to improve the separation performances by developing some free volume in the polymer, as well as to control the porosity (foam structure) and crystallinity (see Figure 11) by facilitating H_2_ transport and reducing the other gases by adsorption/molecular sieving effect, especially for N_2_ [54].

**Figure 14:**
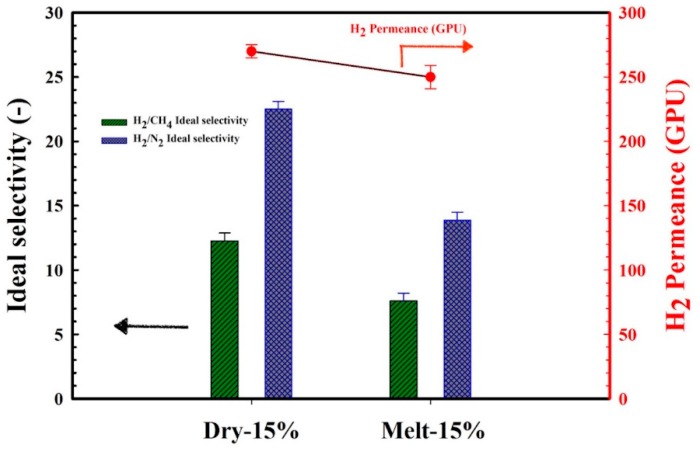
Effect of melt compounding and dry blending on H_2_/CH_4_ and H_2_/N_2_ ideal selectivity and H_2_ permeance of the selected MMFM15 membranes with 15 wt % zeolite.

**Figure 15:**
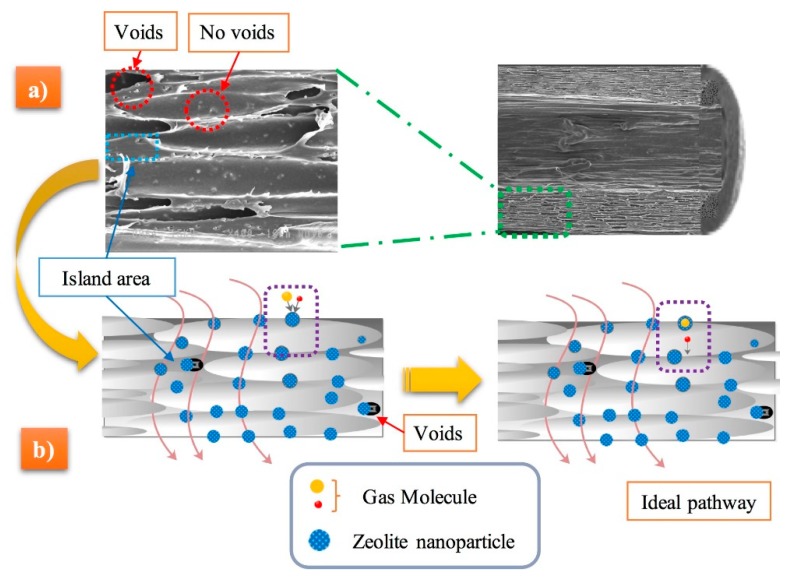
(**a**) Scanning electron microscopy (SEM) of the selected MMFM15 membranes and (**b**) schematic representation of the gas molecule diffusion pathways.

According to Figure 6, it can be seen that the effect of the dry blending and melt compounding techniques on the MMFM15 structure will have a direct effect on the membrane separation performance. Figure 14 clearly shows this effect in terms of gas permeation results. Partial pore blockage of the porous zeolite might be the main reason for the decreasing permeance and selectivity of melt blended membranes [3]. As discussed above, apart from pore blockage, the gas released by the CBA is distributed differently as new gas molecule diffusion pathways are provided by dry blending; this is schematically presented in Figure 15.

### 3.6. Influence of Stretching Speed on the Membranes’ Gas Transport

**Figure 16:**
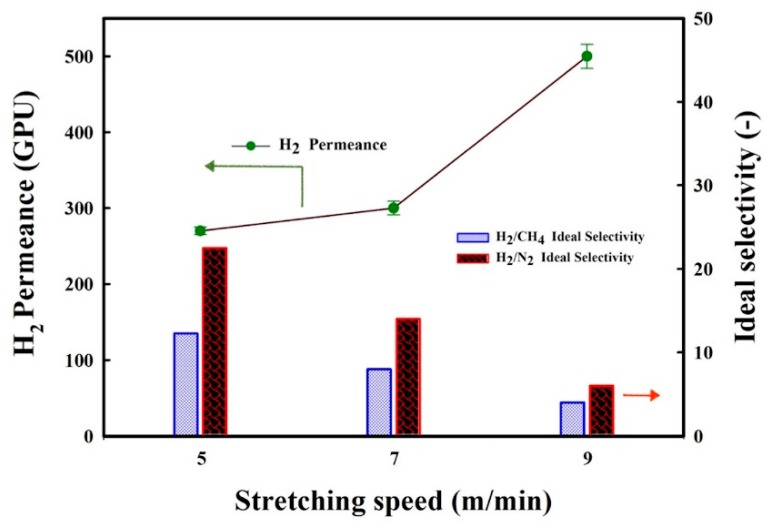
Results for H_2_ gas permeance and H_2_/CH_4_ and H_2_/N_2_ ideal selectivity at 30 °C and 30 psia for MMFM15 at different stretching speeds.

The effect of the stretching speed on the performance of MMFM15 was investigated by preparing membranes at different stretching speed (5, 7, and 9 m/min). The H_2_ permeance and H_2_/CH_4_ and H_2_/N_2_ ideal selectivity of these MMFMs are presented in Figure 16. Increasing the stretching speed significantly increased the H_2_ permeance above 500 GPU, whereas the H_2_/CH_4_ and H_2_/N_2_ ideal selectivity substantially decreased. This implies that some undesirable pores were created between the PE matrix and zeolite particle interface, where the gas molecules might go through (short-circuit). Consequently, the H_2_/CH_4_ and H_2_/N_2_ ideal selectivity significantly decreased below 4. As seen in Figure 15, these interfacial voids shaped mostly in the island area, that is, the area between cells or containing particles, whereas good interfacial adhesion for MMFM15 at 5 m/min was obtained. Based on these observations, the structure produced combined the benefits of both particle addition and stretching [12], as well as foaming to produce multiporous structure membranes to facilitate gas transport properties [7,13].

Zeolite addition to the unfoamed hollow fiber (L-15) membrane led to a negligible variation in separation performance compared to the unfilled and unfoamed hollow fiber (L-0) samples (Figure 13). For H_2_, diffusion more than solubility was responsible for the improved permeance of polyolefins. On the other hand, both CH_4_ and N_2_ solubility and diffusivity were decreased by zeolite addition [26]. Since CH_4_ and N_2_ are larger molecules than H_2_, they move more slowly and/or are trapped inside the cells [13]. Moreover, the incorporation of porous zeolites increased gas adsorption, especially for N_2_. Therefore, the permeance is a function of cell density, zeolite loading, interfacial state, and PE crystallinity.

As a conclusion based on the results obtained, a 70/30/15 blend of LLDPE/LDPE/zeolite with 2.5 wt % of CBA and stretched at 5 m/min seems to be the optimum. To complete the authors’ characterization, mechanical and thermal properties are discussed next.

### 3.7. Mechanical and Thermal Properties

**Figure 17:**
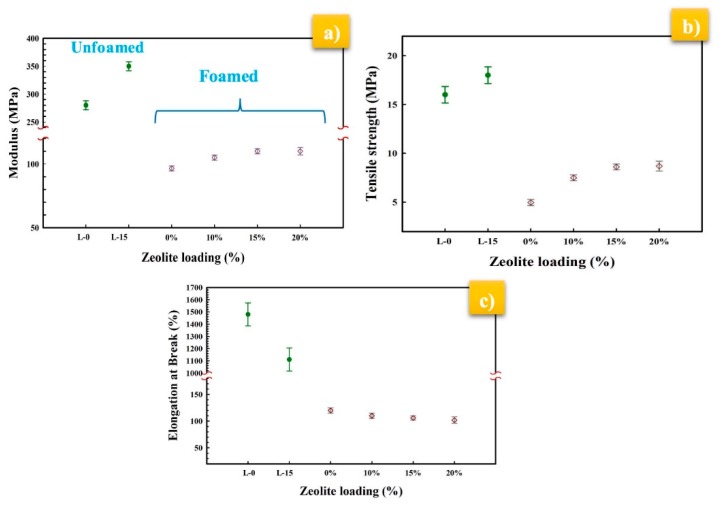
(**a**) Young’s modulus, (**b**) tensile strength, and (**c**) elongation at break of the foamed and unfoamed hollow fibers at different zeolite loading.

The tensile behavior of unfoamed and MMFMs at different zeolite content is shown in Figure 17. Generally, cellular materials have lower modulus and strength when compared to their unfoamed counterpart. This is associated with the fact that less polymer (matrix) was available to sustain the applied stresses. As expected, the tensile modulus and tensile strength of both L-0 (280 MPa, 16 MPa) and L-15 (350 MPa, 18 MPa) (unfoamed samples) are higher than the foamed samples. For the foams, the moduli of MMFM0 to MMFM20 were found to increase with zeolite concentration. Figure 17b shows that the tensile strength trend is similar to the modulus. This is related to the inherent rigid properties of inorganic particles, at improving the mechanical properties of MMFMs for the concentration range studied. On the other hand, the elongation at break of unfoamed samples decreased with zeolite addition (L-0 and L-15), whereas the values for MMFMs decreased less since they are more brittle. These findings are consistent with previous studies [44,55].

**Figure 18:**
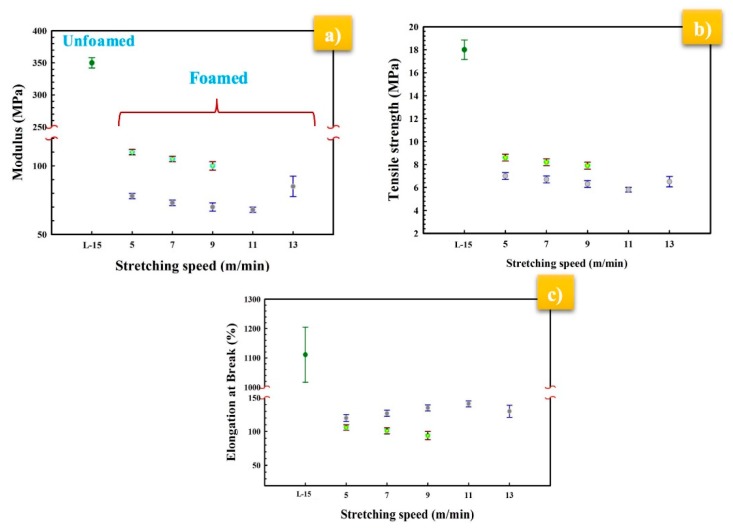
Effect of stretching speed on the (**a**) Young’s modulus, (**b**) tensile strength, and (**c**) elongation at break of selected MMFM15 and L-15.

The effect of the stretching rate on the tensile behavior of the selected MMFM15 membranes is shown in Figure 18a,b. It can be seen that the modulus and strength decreased with increasing stretching rate for both MMFM0 and MMFM15 membranes. However, the elongation at break of MMFM0 increased with increasing stretching speed as seen in Figure 18c, whereas the elongation at break for MMFM15 decreased by increasing the stretching speed due to the presence of zeolite particles.

**Figure 19:**
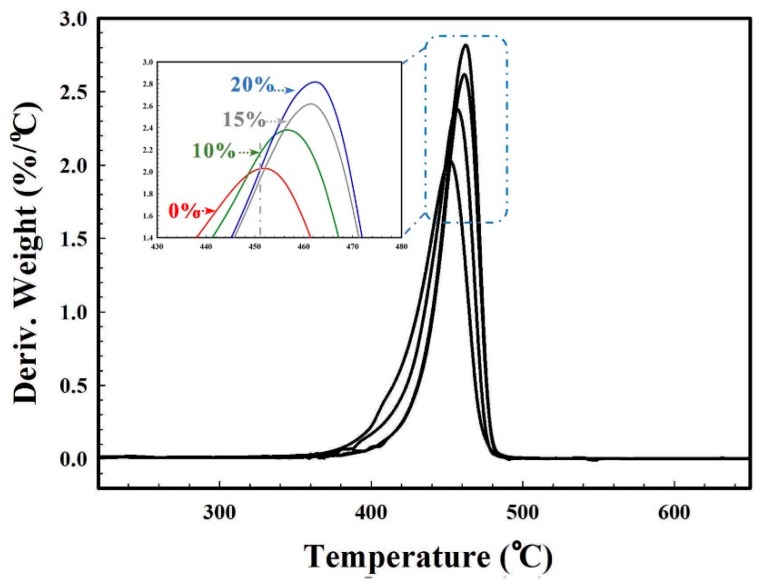
Derivative thermogravimetric analysis (DTGA) curves of MMFM samples compared to unfoamed samples.

Characteristic temperatures from TGA curves are presented in Figure 19. This analysis suggests that the presence of zeolite not only improved the cell size distribution and cell density, but also the membrane thermal stability. Inorganic particles typically have inherently excellent thermal properties compared to polymers. Therefore, it can be seen in the derivative thermogravimetric analysis (DTGA) curves (Figure 19) that the main decomposition temperature (T_max_) significantly increased from 456 to 462 and 463 °C for MMFM10, MMFM15, and MMFM20 membranes, respectively, compared to MMFM0 (451 °C).

**Figure 20:**
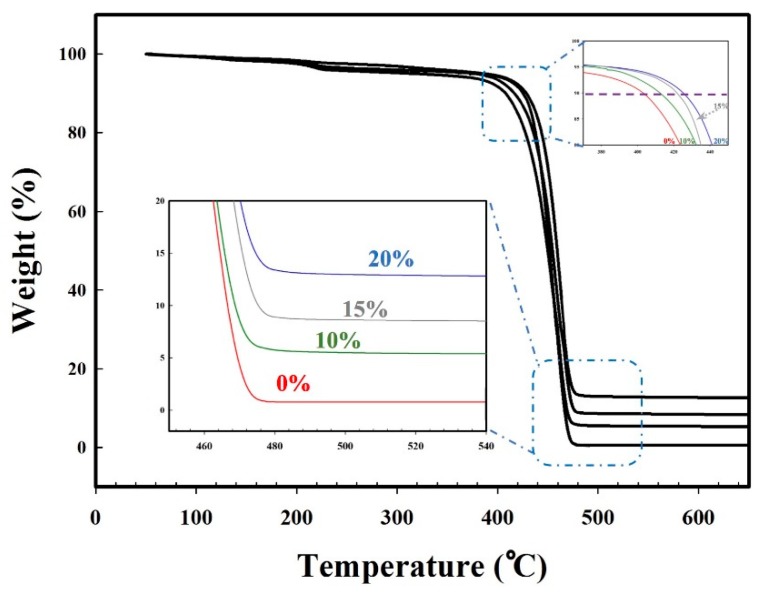
Thermogravimetric analysis (TGA) curves of MMFM samples compared to unfoamed samples.

The effect of zeolite content of the MMFMs has also been studied by TGA (Figure 20). It was found that these membranes are more stable compared to MMFM0. It can be observed that above 475 °C, MMFM0 is totally degraded, whereas the zeolite content (associated with the residues above 500 °C) in MMFM10, MMFM15, and MMFM20 membranes was confirmed to be close to 10%, 15%, and 20%, respectively. Moreover, the values of T10% (the temperature for 10% mass loss) of MMFM0, MMFM10, MMFM15, and MMFM20 are also increasing: 401, 412, 421, and 425 °C, respectively.

## 4. Conclusions

In this work, hollow fiber cellular composite membranes were prepared and optimized from a PE blend (LLDPE/LDPE 70/30) with different zeolite loading (0, 10, 15, and 20 wt %) combined with azodicarbonamide as a chemical blowing agent. The effects of zeolite content as well as stretching rate on the cellular structure and gas separation performance of these mixed matrix foamed membranes (MMFMs) were reported. The membranes were prepared via a fast, cost-effective, and easy continuous extrusion process. SEM images confirmed good zeolite dispersion during manufacturing with this non-solvent technique.

The findings showed that the addition of a porous nanoparticle (zeolite 5A) significantly increased the cell density for the optimum membrane (MMFM15) with a value of 1.2 × 10^9^ cells/cm^3^ and a substantial reduction of cell sizes by approximately 72%. Overall, from a microcellular foaming point of view, the results obtained in this investigation indicate that the zeolites, with their porous structures, have a significant potential to act as nucleating agents for polymer foaming. 

The gas permeation results showed that the H_2_ permeance and H_2_/CH_4_ and H_2_/N_2_ selectivity of the unfoamed (L-0) membranes compared to the optimum membrane (MMFM15) significantly increased from 39 to 270 GPU and 3.3 (H_2_/CH_4_) and 3.9 (H_2_/N_2_) to 12.5 and 22.9, respectively, by introducing zeolite leading to a better cellular structure inside the polyethylene membranes. Furthermore, combining the benefits of both particle addition and stretching, as well as foaming, helped to create multiporous structure membranes to facilitate gas transport properties. Finally, significant permeance and selectivity improvements were obtained compared to the neat matrix.

## Figures and Tables

**Table 1:** Specifications of the hollow fibers produced *.

Unfoamed (Solid)			Foamed			
Sample	Zeolite Concentration (wt %)	Uniaxial Stretching Speed (m/min)	Sample	CBA (wt %)	Uniaxial Stretching Speed (m/min)	Zeolite Concentration (wt %)
L-0	0	5, 7, 9, 11, 13	MMFM0	1.75	5, 7, 9, 11, 13	0
L-10	10	5, 7, 9, 11, 13	MMFM10	2.5	5, 7, 9	10
L-15	15	5, 7, 9, 11, 13	MMFM15	2.5	5, 7, 9	15
L-20	20	5, 7, 9, 11, 13	MMFM20	2.5	5, 7, 9	20

* All unfoamed and foamed samples have a composition of LLDPE/LDPE 70/30 (wt %).
